# Visuo-attentional correlates of Autism Spectrum Disorder (ASD) in children with Down syndrome: A comparative study with children with idiopathic ASD

**DOI:** 10.1016/j.ridd.2020.103678

**Published:** 2020-09

**Authors:** Jennifer M. Glennon, Hana D’Souza, Luke Mason, Annette Karmiloff-Smith, Michael S.C. Thomas

**Affiliations:** a Centre for Brain and Cognitive Development, Department of Psychological Sciences, Birkbeck College, University of London, United Kingdom; b Department of Psychology & Newnham College, University of Cambridge, United Kingdom

**Keywords:** Autism Spectrum Disorder, Down syndrome

## Abstract

**Background:**

Children with Down syndrome (DS) are at increased likelihood of Autism Spectrum Disorder (ASD) relative to the general population. To better understand the nature of this comorbidity, we examined the visuo-attentional processes associated with autistic trait expression in children with DS, focusing specifically on attentional disengagement and visual search performance.

**Method:**

We collected eye-tracking data from children with DS (n = 15) and children with idiopathic ASD (iASD, n = 16) matched according to chronological age. Seven children with DS had a formal clinical diagnosis of ASD (DS+ASD).

**Results:**

In children with iASD, but not DS, higher autistic trait levels were associated with decreased temporal facilitation on a gap-overlap task, implying increased visuospatial orienting efficiency. In all cases, higher autistic trait levels were associated with improved visual search performance according to decreased target detection latency. On a visual search task, children with DS+ASD outperformed their peers with DS-ASD, mirroring the phenotypic advantage associated with iASD. We found no evidence of a relationship between attentional disengagement and visual search performance, providing preliminary evidence of a differentiation in terms of underlying visuo-attentional mechanism.

**Conclusion:**

We illustrate the value of progressing beyond insensitive behavioural measures of phenotypic description to examine, in a more fine-grained way, the attentional features associated with ASD comorbidity in children with DS.

## Highlights

• Visuospatial orienting efficiency increases with symptom severity in idiopathic ASD.
• Children with Down syndrome and comorbid ASD display superior search performance.
• Visuo-spatial orienting ability and visual search performance appears unrelated.

## What this paper adds?

There is a growing narrative in the literature of a distinct profile of Autism Spectrum Disorder (ASD) in children with Down syndrome (DS) relative to children with isolated or idiopathic ASD (iASD). This carries clinical implications with regard to the validity of ASD diagnosis and the utility of current intervention programmes in DS populations. The research from which this narrative has developed has focused primarily on behavioural measures (e.g., parent-report questionnaires) that are arguably insensitive to varying forms of social and communicative difficulty. We designed an eye-tracking study to examine the visuo-attentional processes associated with ASD comorbidity in children with DS relative to children with DS and no ASD (DS-ASD) and children with iASD. We found that children with DS + ASD were better at visual search than their peers with DS-ASD, mirroring the performance profile associated with iASD. This suggests that, in some ways, comorbid presentations of ASD in children with DS are similar to idiopathic forms of ASD.

On the gap-overlap task of visuo-spatial orienting, we found that the performance profile observed in children with iASD was distinct when compared to children with DS with or without comorbid ASD. This suggests that in other ways, presentations of ASD in DS may differ in terms of underlying visuo-attentional mechanism. This study represents an important first step in the characterisation of ASD in DS according to performance on eye-tracking measures of visuo-attentional ability.

## 1 Introduction

Autism Spectrum Disorder (ASD) is a clinical umbrella term used to reference a profile of socio-communicative impairment and restricted, repetitive patterns of behaviour (RRB; Diagnostic and Statistical Manual of Mental Disorders; DSM-5; American Psychiatric Association, 2013). Most cases of ASD are considered multi-genic meaning that they arise from the cumulative impact of many common genetic risk variants (De Rubeis & Buxbaum, 2015; Ramaswami, 2018). In 10 % of cases, ASD coincides with genetic syndromes of known aetiology (Cook & Scherer, 2008; Gai et al., 2012). Down syndrome (DS) is the most frequently occurring of these genetic syndrome groups. Caused by a trisomy of chromosome 21, DS presents in one in every 700 live births and is associated with increased risk of ASD (Parker et al., 2010); a significant minority of individuals (approximately 18 %) reach screening thresholds for ASD (DiGuiseppi et al., 2010; Moss, Richards, Nelson, & Oliver, 2013; Richards, Jones, Groves, Moss, & Oliver, 2015).

Reports of elevated ASD prevalence in DS populations have sparked considerable debate concerning the precise nature of the observed socio-communicative difficulties and RRBs. Empirical enquiry into the behavioural profiles associated with ASD in children and adults with DS has revealed subtle differences relative to idiopathic ASD (iASD) comparison groups (for a review, Glennon, Karmiloff-Smith, & Thomas, 2017). Hepburn, Philofsky, Fidler, and Rogers (2008) conducted a longitudinal examination of autistic trait expression in toddlers with DS. They found that difficulties in communication and play were accompanied by a number of developmentally appropriate social skills that included sharing, engaging in joint attention and directing vocalisations to others. Moss et al. (2013) examined phenotypic presentations according to ASD symptomatology in adults with DS using the Social Communication Questionnaire (SCQ; Rutter, Bailey & Lord, 2003) and in adolescents with iASD who were matched according to symptom severity and level of adaptive functioning. They reported broadly similar phenotypic profiles. However, ASD in DS was associated with less environmental withdrawal suggesting subtle differences in the presentation of autistic-like traits. Similarly, Warner, Moss, Smith, and Howlin (2014) examined behavioural presentations of ASD comorbidity in 6- to 15-year olds with DS relative to a reference sample of individuals with iASD. Despite reaching the SCQ screening threshold for ASD, the data revealed that children and adolescents with DS were significantly less likely to show impairment in several aspects of non-verbal communication including the use of gesture and imitation. They were also significantly less likely to demonstrate impairment on items corresponding to social exchange and reciprocity. The authors hypothesised that relatively high levels of social motivation in DS may function as a protective factor against the socio-communicative difficulties typically observed in children with iASD (Fidler, Hepburn, & Rogers, 2006; Kasari & Freeman, 2001; Loveland & Kelley, 1991; Rosner, Hodapp, Fidler, Sagun, & Dykens, 2004).

As DS is the most common chromosomal cause of intellectual disability, it has been suggested that cognitive factors play a role in the emergence and expression of comorbid ASD. Skuse (2007) proposed that intellectual disability might diminish the brain’s capacity to compensate for the presence of independently inherited genetic risk variants, thereby facilitating the manifestation and development of autistic-like traits. In terms of underlying mechanism, a deficit in neuronal network connectivity has been proposed to account for increased ASD risk in low functioning populations (Dierssen & Ramakers, 2006; Geschwind & Levitt, 2007). There have been various reports of a negative association between ASD symptom severity and indices of intellectual ability in children with DS (DiGuiseppi et al., 2010; Molloy et al., 2009). However, it is worth noting that not all genetic syndrome groups characterised by intellectual disability feature high rates of ASD (Moss & Howlin, 2009). Moreover, in the case of DS, children with comorbid diagnoses of ASD have been found to exhibit significantly elevated ASD trait scores above and beyond the variance accounted for by differences in intellectual functioning (Molloy et al., 2009). While cognitive ability plays a clear role in phenotypic expression, it does not appear to account in full for the heightened prevalence of autistic-like traits in DS (Lee, Martin, Berry-Kravis, & Losh, 2016). The question remains: is this a similar form of ASD to that which is observed in cases of iASD? To address this question, we look to the attentional processes underlying this complex comorbidity, moving beyond behavioural measures of phenotypic description that are often limited in their sensitivity and specificity when applied to individuals with genetic syndromes associated with intellectual disability (Moss et al., 2009).

Attention is the means through which we selectively perceive and process, with an aim to navigating, our external worlds and spatial orienting is the shifting of attention between targets in a visual field (Desimone, Robert & Duncan, 1995). This involves three discrete operations: disengaging, shifting, and re-engaging attention (Posner & Petersen, 1990; Posner, Walker, Friedrich, & Rafal, 1984). Attentional disengagement difficulties have been frequently reported in children with iASD and have, in longitudinal studies, been found to precede the expression of the phenotype in infants at high familial risk of developing the condition (Elsabbagh et al., 2009; Kleberg, Thorup, & Falck-Ytter, 2017; Landry & Bryson, 2004; Zwaigenbaum et al., 2005; but see, Johnson, Lum, Rinehart & Fielding, 2016). This is significant because any disruption or delay to the maturation of visual and attentional brain systems and, subsequently, to an infant’s ability to orient flexibly is likely to directly impact their socio-communicative development (Johnson, 2001).

Visual search is another task domain in which visuo-spatial orienting in individuals with iASD manifests atypically (for reviews, see Dakin & Frith, 2005; Simmons et al., 2009). Visual search performance is often indexed according to the time it takes the viewer to locate target items amidst distractor stimuli (Treisman & Gelade, 1980). Superior search performance (i.e., reduced target detection latency) has been implicated in the emergence and early phenotypic expression of iASD (Cheung, Bedford, Johnson, Charman, & Gliga, 2018; Gliga, Bedford, Charman, Johnson, & BASIS Team, 2015) and has been frequently observed in older children with iASD relative to neurotypical controls (Jarrold, Gilchrist, & Bender, 2005; Kaldy, Kraper, Carter, & Blaser, 2011; Plaisted, O’Riordan, & Baron-Cohen, 1998). Theoretical interpretations of this phenotypic advantage posit that iASD is characterised by anomalies in the top-down modulation of visuo-perceptual inputs. Weak central coherence (Happé & Frith, 2006) and enhanced perceptual functioning (Caron, Mottron, Berthiaume, & Dawson, 2006) models maintain that decreased target detection speeds are due, at least in part, to a local processing bias. This is the proposed tendency for individuals with iASD to preferentially process the local featural properties of a stimulus over its global form. Alternatively, superior search performance in children with iASD has been theorised to emerge on account of an irregular alerting system (Keehn, Müller, & Townsend, 2013). As originally described by Posner and Petersen (1990), this system is responsible for achieving and maintaining a homeostasis in terms of sensitivity/arousal levels in response to incoming sensory information. Liss, Saulnier, Fein, and Kinsbourne (2006) propose that early irregularities in the development of this system result in an excessively focused attentional style which facilitates superior processing of stimulus features at the locus of attention, manifesting as superior visual search performance in infants and children with iASD.

Previously, indices of attentional disengagement and visual search performance have been studied in isolation (i.e., not in relation to one another). It seems counterintuitive that children with iASD may be slower to disengage and shift attention, but quicker to serially search for target items in visual search arrays. However, Keehn et al. (2013) offer a theoretical account of phenotypic emergence that endeavours to bridge this apparent dichotomy. They propose that basic-level deficits in visuo-spatial orienting are a potential means through which an infant’s ability to self-regulate is disrupted. This was based on observations that typically developing infants self-regulated their arousal levels by intermittently disengaging and shifting their gaze away from faces that were present in their visual field (Field, 1981). Keehn et al. (2013) suggest that early disengagement difficulties may prompt a compensatory attentional narrowing as the system endeavours to self-regulate arousal by reducing the amount of incoming information. Further, they propose that this compensatory narrowing may account for the visual search advantage often observed in children with iASD as an increased signal-to-noise ratio may generate an enhanced capacity to process stimulus features at the locus of attention (for reviews, see Dakin & Frith, 2005; Simmons et al., 2009). This theoretical account of phenotypic emergence yields a testable hypothesis in reference to the current study: if both visuo-attentional phenotypic features share a common underlying mechanism, such as an increased signal-to-noise ratio,performance on visual search tasks will vary according to indices of attentional disengagement efficiency. A primary objective of this study, then, is to look at the relationship between attentional disengagement and visual search performance in a cohort of children with iASD.

While irregularities in attentional disengagement and visual search performance have been implicated in idiopathic forms of ASD, it remains to be seen whether autistic-like traits in children with DS are associated with these same characteristics. A second main objective of the current study is to examine performance on eye-tracking tasks designed to measure attentional disengagement and visual search performance in children with DS with ASD (DS + ASD) and DS without ASD (DS-ASD).

Visuo-attentional abilities have been examined previously in children with DS, though not in relation to ASD comorbidity. Relative to mental age-matched neurotypical controls, infants and children with DS have been found to take significantly longer to search for target items on visual search tasks (Munir, Cornish, & Wilding, 2000; Steele, Brown, & Scerif, 2011). Attentional orienting ability, according to performance on a double-step saccade task, has been examined in infants with DS (Gilmore & Johnson, 1995; Steele et al., 2011). According to the data, infants with DS performed similarly to mental age-matched neurotypical controls in terms of their ability to disengage and orient attention in response to the onset of secondary visual stimuli. This finding adhered to previous reports of developmentally appropriate visual orienting abilities in adults (Randolph & Burack, 2000) and adolescents with DS (Goldman, Flanagan, Shulman, Enns, & Burack, 2005).

In sum, we know little of the attentional features associated with presentations of socio-communicative difficulty and RRB in children with DS. In terms of clinical relevance, there is ongoing uncertainty surrounding the nature and validity of ASD in children with DS, often resulting in prolonged diagnostic decision making and delayed access to intervention services. Empirical efforts to elucidate the precise nature of autistic-like traits in DS are necessary to inform and improve the clinical management of those who reach diagnostic thresholds for ASD, and there is need to do so; higher levels of autistic-like impairment predict poorer prognostic outcomes in individuals with DS (Carter, Capone, Gray, Cox, & Kaufmann, 2007; DiGuiseppi et al., 2010; Warner et al., 2014). In order to better understand the nature of the autistic-like traits in DS, fine-grained analysis of their attentional correlates is required.

In this study, we examined attentional disengagement and visual search performance in children with low-functioning iASD and in children with DS +/- ASD. Firstly, we hypothesised that children with iASD and DS + ASD would find it difficult to disengage and shift attention when presented with competing visual stimuli relative to cases of DS-ASD; this is often termed ‘sticky attention’. Secondly, we predicted superior search performance according to reduced target detection latency in children with iASD and DS + ASD relative to those with DS-ASD. Finally, in all cases and in keeping with the notion of a common underlying mechanism, we anticipated a relationship between indices of attentional disengagement and visual search performance.

## 2 Method

### 2.1 Participants

A total of 16 children with iASD and 15 children with DS took part in this study. Ethical approval was obtained from the Birkbeck Department of Psychological Sciences. Children formally diagnosed with iASD were recruited through the ASD–UK Foundation. Children with DS were recruited with the support of the Down Syndrome Association, a registered UK-based charity organisation. Exclusion criteria were a clinical history of epilepsy and previous incidences of acquired brain injury. Participants were categorised into three groups: iASD, DS-ASD and DS + ASD. Participants were classified as DS + ASD if they had received formal clinical diagnoses of both conditions prior to testing. The presence or absence of ASD was verified according to the results of the Autism Diagnostic Observation Schedule (second edition; ADOS-2) which was administered to all participants on the day of testing (Lord et al., 2011, 2012). Cohen’s kappa values were generated to look at the agreement between ADOS-2 outputs and the clinical provision of ASD diagnoses. There was substantial agreement (.96) between clinical diagnostic status and ADOS-2 cut-off data within the complete dataset; κ = .73, p < .001. Agreement within the iASD and DS cohorts was .93 an .87, respectively. Groups were age-matched but differed according to non-verbal intellectual ability, as determined by the Leiter International Performance Scales, third edition (Leiter-3; Roid, Miller, Pomplun, & Koch, 2013), and autistic trait severity, as indexed by the Social Responsiveness Scale, second edition (SRS-2; Constantino & Gruber, 2012; see Table 1).

**Table 1:** Demographics Data by Group with ANOVA Outputs.

	**iASD** (*n* = 16)		**DS + ASD** (*n* = 7)		**DS-ASD** (*n* = 8)			
Gender (*m/ f)*	16/0		4/3		4/4			
	*M*_(_*_SD_*_)_	Range	*M*_(_*_SD_*_)_	Range	*M*_(_*_SD_*_)_	Range	**Sig.**	**Post-hoc**
Age _years_	8.5 _(1.6)_	5.8 - 10.8	8.7 _(1.8)_	6.8 - 11.2	9.1 _(2.2)_	6.3 - 12	.765	---
Leiter-3^a^	56 _(22)_	17–91	30 _(18)_	06–55	49 _(15)_	19 - 65	.022	iASD > DS + ASD
SRS-2^b^	77 _(13)_	55−90	78 _(12)_	59−90	57 _(7)_	48−69	.001	iASD = DS + ASD > DS-ASD

a Note: Higher raw Leiter-3 scores index increased intellectual ability.

b Note: Higher total SRS-2 scores index increased autistic trait severity.

### 2.2 Measures and procedure

Data collection took place at the Birkbeck Babylab in London. Prior to all testing sessions, parents/caregivers of participants were briefed and written participatory consent was acquired. Participants completed a behavioural assessment which included the ADOS-2 and the Leiter-3, and an eye-tracking session which featured gap-overlap and visual search tasks. An additional measure of autistic trait expression – the SRS-2 - was administered to the parents/caregivers of all participants. Each of these measures is detailed subsequently.

#### 2.2.1 Autism diagnostic observation schedule, second edition

The ADOS-2 is a semi-structured standardised assessment of autistic symptomology (Lord, Luyster, Gotham, & Guthrie, 2011; Lord, Rutter, DiLavore, Risi, Gotham, & Bishop, 2012). It was administered to all participants as a means to verify the presence or absence of ASD. There are five module options, each designed to cater for different chronological ages and different levels of expressive language ability. Participants in the current study received modules 1 or 2. They were engaged in a variety of play-based activities to elicit developmentally-appropriate signatures of social ability and RRB.

#### 2.2.2 Leiter international performance scales, third edition

Non-verbal intelligence was indexed according to the Leiter-3, a standardised measure intended for use with individuals with ASD and intellectual disability (Roid et al., 2013). A cognitive battery of four subtests was administered. These were: (1) figure ground (i.e., identifying embedded figures within complex pictorial stimuli), (2) form completion (i.e., recognising ‘whole objects’ from fragmented visual representations, (3) classifications/ analogies (i.e., object and/or geometric design classification), and (4) sequential order (i.e., pattern completion). Total raw scores were computed by adding together participant’s four sub-test values, with higher scores indexing increased non-verbal intellectual ability.

#### 2.2.3 Social responsiveness scale, second edition

The SRS-2 is a 65-item parent-report questionnaire that provides a total score index of autistic-like symptomology; scores in the range of 60 and 65 signal mild to moderate trait levels while scores of 66 and higher signal clinically significant levels of symptomology (Constantino & Gruber, 2012). Parents/caregivers of participants aged 4 years and above received the school-aged version of the questionnaire, while parents/caregivers of younger children received the pre-school version of the questionnaire. Both provide total scores on a similar scale so questionnaire outputs may be considered and analysed together.

#### 2.2.4 Eye tracking

All participants were engaged in a 12-minute eye-tracking session. They were seated in front of a 23-inch liquid-crystal display (LCD) monitor at a distance of 60 cm and verbally instructed to sit comfortably, pay close attention to the ‘television’ and try their best not to move or wiggle around. As required, efforts would be made to encourage continued engagement from the participating children such as issuing verbal praise (e.g., “you’re doing so well”, “great job”) or allowing children to take a programmed break halfway through the eye-tracking session.

All stimulus presentation and data collection were performed using the Task Engine (https://sites.google.com/site/taskenginedoc/) framework for Matlab (MathWorks, Inc., Natick, Massachusetts, United States). An automatic five-point calibration was run wherein coloured spirals expanded and contracted in each of the four corners and in the centre of the screen. Following this brief five-point calibration, participants completed a gap-overlap task (adapted from Elsabbagh et al., 2013; Landry & Bryson, 2004) and a visual search task (adapted from Kaldy et al., 2011; Treisman & Gelade, 1980). Data were collected using a Tobii TX300 eye-tracking system (Tobii AB, Stockholm, Sweden). A webcam was used to monitor behaviour and sessions were video recorded for analytic reference.

##### 2.2.4.1 Gap-overlap task

For the gap-overlap task, participants were presented with a gaze-contingent central stimulus (CS, i.e., a dynamic colourful clock) and, subsequently, a peripheral stimulus (PS, i.e., a white cloud) which appeared randomly either to the right or the left of the CS at an eccentricity of 19°. According to real-time eye-tracking data, the CS initially loomed on screen until it was fixated upon which immediately triggered a spinning of the CS for a random 600−700 ms to ensure attentional engagement and the onset of the PS. When the participant fixated upon the PS, or 2.5 s after PS onset (whichever was sooner) the reward stimulus was presented. This took the form of various cartoon characters at the location of the PS which span or shrank whilst emitting various child-friendly sounds. Saccadic reaction times (SRTs) were calculated as the latency in ms to disengage from the CS and saccade to the PS.

Attentional disengagement was probed in three conditions which manipulated the timing and order of the relative onset and offset of the CS after PS onset. In the baseline condition, CS offset occurred at the same time as PS onset. In the gap condition, CS offset preceded PS onset by 200 ms. In the overlap condition, the CS remained on screen at PS onset and for the duration of PS presentation. Over 60 trials in five blocks of 12, we this arrived at an average SRT for each participant, for each condition. We then computed difference scores measuring disengagement (DIS), formed by calculating the mean SRT difference between overlap and baseline trials, and facilitation (FAC) by calculating the mean SRT difference between baseline and gap trials.

A minimum of 6 valid trials per condition was necessary in order for data to be retained in subsequent analyses. Trials were considered valid according to several criteria: (1) data quality was acceptable to form SRT estimates; (2) there were no periods of missing data greater than 200 ms following central fixation or 50 ms on either side of the peripheral stimulus onset; (3) gaze did not move in the opposite direction after leaving the central stimulus, and (4) SRT was between 150 ms and 1200 ms. Any trials in which these criteria were not met were excluded from subsequent analysis.

##### 2.2.4.2 Visual search

The visual search task involved presenting participants with search arrays with a verbal instruction to “find the red apple”, the target stimulus, amidst distractor stimuli (i.e., blue apples and red rectangles). The target was always present. There were 12 search trials in total featuring set sizes 5, 9 or 13 in equal amount and in random order. Participants were presented with a central fixation point prior to the onset of each trial. For each trial, the task computed our variable of interest which was the latency to visually locate and fixate upon the search targets. Search arrays were presented for a maximum duration of 4 s or until the target stimulus (i.e., the red apple) was fixated upon.

This was a conjunction search task (i.e., target and distractor items shared a conjunction of features) as opposed a single feature search task wherein the target item can be identified according to a single feature dimension. Conjunction search trials have been found to be more sensitive than single feature search paradigms at eliciting iASD and non-iASD group differences in performance (e.g., Kaldy et al., 2011; O’Riordan, 2000; O’Riordan, Plaisted, Driver, & Baron-Cohen, 2001; Plaisted et al., 1998). Further, single feature search performance plateaus by approximately 2 years of age, typically, while conjunction search performance continues to develop throughout childhood and adolescence (Brennan, Bruderer, Liu-Ambrose, Handy, & Enns, 2017; Donnelly et al., 2007; Woods et al., 2013). Considering the chronological age and developmental level of our participants, it was deemed more appropriate to examine conjunction search performance in the current study.

### 2.3 Planned statistical analyses

Data were plotted and analysed using Microsoft Excel and IBM SPSS software (version 24). Analyses of variance (ANOVAs) and independent samples *t*-tests were employed to assess mean group differences in attentional disengagement and visual search performance. Autistic trait variation, as indexed by the SRS-2, was analysed within and between groups according to mean SRT data derived from the gap-overlap task and mean target detection latency on visual search trials. To do this, we used a trajectory analysis approach (Thomas et al., 2009). This method is akin to standard analyses of covariance (ANCOVA) but instead of testing group mean differences, performance is examined in relation to linear intercepts and gradients. Main and interaction terms were entered manually and for each analysis, the x-axes were re-scaled to ensure all main effects were calculated at the first point of overlap. In cases of multiple comparisons, we considered statistically significant effects against a Bonferroni corrected significance level.

Prior to applying this approach to statistical analysis, we checked that the data adhered to a number of testing assumptions. Shapiro-Wilks tests were run to check that data were normally distributed. The results revealed a significant positive skew for mean SRT data on overlap trials. A log10 transformation was applied to improve the distribution of these data for analysis. Graphical illustrations of inferential outputs and references to raw data present overlap SRTs as they were pre-transformation. We ran Levene’s Test for Equality of Variances to confirm homogeneity of variance for all variables of interest.Following this, we used linear regression modelling to check that the data conformed to the test assumptions of linearity and heteroscedasticity. Visual inspection of P-P plots confirmed linearity in all cases. Similarly, visual inspection of scatterplots of predicted SRS-2 scores against standardised residuals for variables of interest confirmed homoscedasticity in all cases.

## 3 Results

### 3.1 Performance on gap-overlap task according to group

**Fig. 1:**
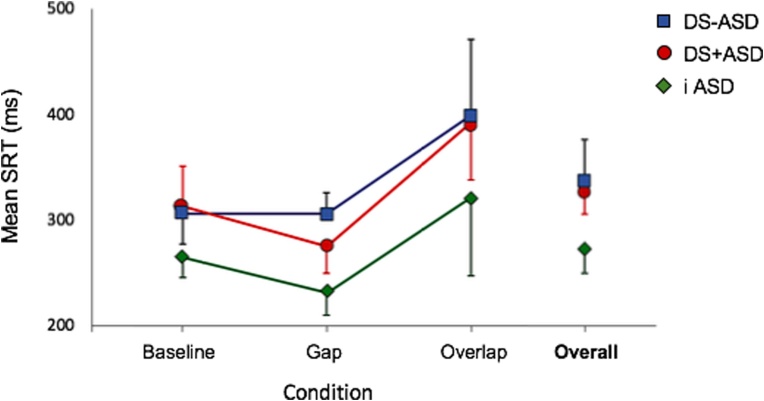
Mean saccadic reaction time (SRT) data by group per gap-overlap condition. Error bars represent 95 % confidence intervals.

Our first step was to confirm that our gap-overlap paradigm functioned as we would expect it to. A mixed 3 × 3 ANOVA was run to examine within- and between-group (iASD, DS-ASD and DS + ASD) differences in SRT data derived from each gap-overlap condition (gap, baseline and overlap). There was a significant main effect of condition; *F* (1.4, 28.6) = 15.82, *p* < .001, *η_p_^2^* = .43.^1^ According to pairwise comparisons, participants took longer to disengage and shift attention on overlap trials (*M* = 354, *SD* = 106) relative to gap (*M* = 260, *SD* = 54; *p* < .001) and baseline trials (*M* = 285, *SD* = 56; *p* = .007). Additionally, mean SRTs were significantly reduced on gap relative to overlap trials (*p* < .001). With regard to a main effect of group, there was a trend level of significance; *F* (2,21) = 3.46, *p* = .050, *η_p_^2^* = .25. For reference, this reflected an overall SRT increase of 64 ms in children with DS-ASD relative to children with iASD. There was no condition × group interaction effect; *F* (4,42) = 0.31, *p* = .871, *η_p_^2^* = .03 (see Fig. 1) which means that FAC and DIS did not differ significantly across groups.

### 3.2 Relating gap-overlap performance to autistic trait severity

Next, we assessed variability in SRS-2 scores according to performance on the gap-overlap task and examined whether the relationship was modulated by group. As we were interested in the full range of SRS-2 scores within the complete DS cohort, participants were split according to group with two levels (iASD and DS). First, we wanted to know if these two participant groups varied significantly in terms of autistic trait expression according to performance on the gap-overlap task, specifically in reference to the size of the SRT difference between baseline and overlap trials (i.e., DIS). A modified ANCOVA was run to examine within and between-group variation in SRS-2 scores according to DIS. Total SRS-2 scores were the dependent variable, DIS was entered as a covariate and the fixed factor was group (iASD and DS). The interaction term (group × DIS) was also included in the model. There was no main effect of DIS [*F* (1, 19) = 0.01, *p* = .985, *η_p_^2^* = .001], no main effect of group [*F* (1, 19) = 0.02, *p* = .894, *η_p_^2^* = .001] and no group × DIS interaction effect [*F* (1, 19) = 4.61, *p* = .045, *η_p_^2^* = .20].

**Fig. 2:**
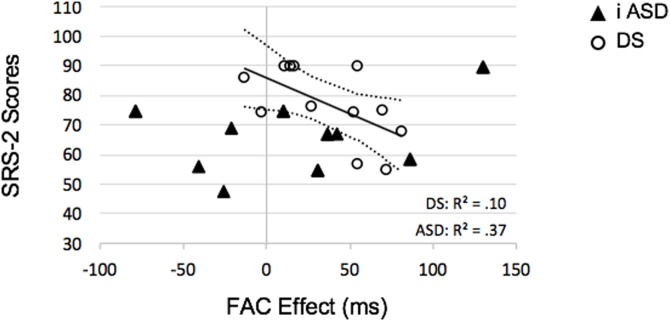
Total SRS-2 scores plotted against mean FAC effect sizes for iASD and DS cohorts. Confidence intervals (95 %) are attached to the iASD trajectory for reference.

Next, we wanted to know if SRS-2 scores in participant groups varied significantly according to SRT difference between baseline and gap trials (i.e., FAC). A modified ANCOVA was run to examine within- and between-group variation in SRS-2 scores according to FAC. Total SRS-2 scores were the dependent variable, FAC was entered as a covariate and the fixed factor was group (iASD and DS). The interaction term (group × FAC) was also included in the model. There was no main effect of FAC [*F* (1, 19) = 2.04, *p* = .169, *η_p_^2^* = .10], but there was a significant main effect of group [*F* (1, 19) = 9.86, *p* = .005, *η_p_^2^* = .34] and a significant group × FAC interaction effect [*F* (1, 19) = 6.21, *p* = .022, *η_p_^2^* = .25]; higher SRS-2 scores were associated with smaller FAC effect sizes in children with iASD (*R* = −.61, *p* = .036) but not in children with DS (*R* = .32, *p* = .338; see Fig. 2

To confirm that there was no effect of chronological age, we ran bivariate correlation analyses. No significant correlations emerged to suggest a relationship between FAC and chronological age, or total SRS-2 scores and chronological age, in either participant cohort.

### 3.3 Visual search performance according to group

**Fig. 3:**
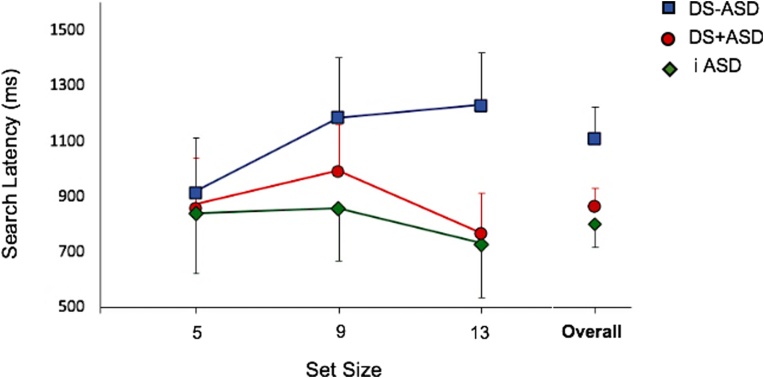
Mean search latencies by group as a function of set size. Error bars represent 95 % confidence intervals.

To test the hypothesis that children with iASD and DS + ASD would exhibit superior search performance relative to children with DS-ASD, we examined mean target detection latency by set size (5, 9 and 13) and group (iASD, DS-ASD and DS + ASD). A mixed 3 × 3 ANOVA was run. Fig. 3 presents the mean target detection latencies as a function of set size by group. There was no significant main effect of set size [*F* (2, 54) = 0.77, *p* = .470, *η_p_^2^* = .03] but there was a significant main effect of group [*F* (2, 27) = 5.35, *p* = .011, *η_p_^2^* = .28]. No group × set size interaction effect was observed [*F* (4, 54) = 0.67, *p* = .616; *η_p_^2^* = .05].

To test our a priori hypothesis that children with DS + ASD would outperform their peers with DS-ASD, we ran an independent samples *t*-test and found a significant group difference in conjunction search latency overall; *t* (13) = 3.07, *p* = .009, Cohen’s *d* = 3.2. Of note, this significant group difference remained when non-verbal intellectual ability, as indexed by raw Leiter-3 scores, were controlled for in an ANCOVA. Additionally, pairwise comparisons revealed significantly increased search latencies overall in children with DS-ASD relative to those with iASD (*p* = .010).

### 3.4 Relating visual search performance to autistic trait severity

Next, we assessed variability in SRS-2 scores according to visual search by group. Again, we were interested in the full range of SRS-2 scores within the complete DS cohort, so participants were split according to group with two levels (iASD and DS). A modified ANCOVA was conducted to examine within and between-group variation in total SRS-2 scores according to search latencies. Total SRS-2 scores were entered as the dependent variable. The fixed factor was group (iASD and DS) and the co-variate was mean search latency. The interaction term (group × mean search latency) was included in the model.

There was no main effect of group [*F* (1, 26) = 0.13, *p* = .723, *η_p_^2^* = .01] but there was a significant main effect of search latency reflecting an overall trend in the dataset: children with higher SRS-2 scores were quicker to locate target items [*F* (1, 26) = 8.04, *p* = .009, *η_p_^2^* = .24]. No significant interaction effect emerged [*F* (1, 26) = 0.09, *p* = .771, *η_p_^2^* = .003].

To confirm that there was no effect of chronological age, we bivariate correlation analyses. No significant correlations emerged to suggest a relationship between mean conjunction search latency (collapsed across all set sizes) and chronological age in either participant cohort.

### 3.5 Attentional disengagement and visual search performance

Finally, we were interested in whether the eye-tracking variables that yielded significant outputs (i.e., FAC and mean search latency) contributed independently to variability in phenotypic expression according to total SRS-2 scores in children with iASD. First, we ran a bivariate correlation analysis to examine the relationship between FAC and mean search latency within the iASD cohort; no significant correlation emerged (*R* = .31, *p* = .307). Next, we ran a multiple linear regression to predict SRS-2 scores according to FAC and mean search latency in children with iASD. The regression model was significant [*F* (2, 9) = 5.38, *p* = .029, *R*^2^ = .545] but only FAC independently accounted for a significant proportion of the variability in total SRS-2 scores (see Table 2).

**Table 2:** Summary of Hierarchical Multiple Regression Analysis for Variables predicting SRS-2.

SRS-2	*B*	*SE*	*β*	*t*	*p*
FAC	−.208	.091	−.522	−.2.276	.049
Search latency	−.020	.011	−.427	−1.861	.096

## 4 Discussion

We set out to investigate whether ASD in DS is characterised by the same visuo-attentional features that have been documented in cases of iASD, relating to a broader question of whether these are similar clinical entities in accordance with current classification and diagnostic practices. In terms of the SRT data derived from the gap-overlap task, we found no evidence of increased attentional disengagement difficulty in children with iASD and DS + ASD relative to those with DS-ASD. This is inconsistent with the results of Landry and Bryson’s (2004) study wherein a gap-overlap task was administered to 5-year-olds with iASD and DS who were matched according to chronological age and intellectual ability. According to their data, children with iASD took significantly longer to disengage and shift attention on overlap trials relative to their peers with DS. The authors interpreted this result as signalling a degree of syndrome-specificity as iASD, but not DS, was associated with ‘sticky attention’ in contexts of competing visual stimuli. In reference to the contrasting results of the current study, variations in task design and methodology warrant consideration; in particular, the degree to which the stimuli employed in any gap-overlap task are salient to children with ASD is likely to influence the sensitivity of the paradigm. Landry and Bryson’s (2004) stimuli consisted of dynamic geometric imagery and since then, a visual preference for repetitive motion pertaining to geometric stimuli has been documented in children with idiopathic ASD (Pierce, Conant, Hazin, Stoner, & Desmond, 2011, 2016). Additionally, age-related differences warrant consideration; our participant samples were older. iASD in early childhood may be marked by longer SRTs on overlap trials due to a delay in the development of corresponding oculomotor control systems; similar SRTs in older children with iASD and DS may be indicative of a developmental catch-up following this initial period of delay and/or a reduced sensitivity of overlap trials to differentiate clinical cohorts with increasing chronological age.

Next, we examined variability in autistic-like trait expression according to SRT on the gap-overlap task. We found no evidence of a relationship between DIS and SRS-2 scores for either participant cohort, adding to a growing literature which suggests that disengagement difficulties in contexts of competing visual stimuli, often termed ‘sticky attention’, may not be as robust a marker of idiopathic ASD as previously implied (Fischer, Koldewyn, Jiang, & Kanwisher, 2014, 2016; van der Geest, Kemner, Camfferman, Verbaten, & van Engeland, 2001; Wilson & Saldaña, 2018). Rather, our results showed that the current iASD and DS cohorts differed significantly with regard to variability in autistic trait severity according to FAC; smaller FAC effect sizes were associated with increased SRS-2 scores in children with iASD but not in children with DS. This mirrors, to some extent, a study by Wilson and Saldaña (2018) who administered the gap-overlap task to 7-year-old children with iASD and chronological age-matched neurotypical controls; no significant group differences were observed on trials characterised by competing visual stimuli (i.e., overlap trials). Rather, those with idiopathic ASD were differentiated from their neurotypical peers in demonstrating significantly decreased SRTs on gap trials. Typically, a reduction in FAC is considered an index of the maturity and efficiency of corresponding visual and attentional brain systems (Johnson, 1990; Johnson & Tucker, 1996). It may therefore be the case that increased phenotypic expression in terms of behavioural symptomatology corresponds to increased visuo-spatial orienting efficiency, as reflected in a reduced FAC effect size, in iASD. In congruence with this notion, studies have found increased activation of the frontal eye fields and elevated functional connectivity between frontal and occipital brain regions in children with iASD (Keehn et al., 2008, 2013). In DS, conversely, no significant association emerged between FAC and autistic trait severity, implying a phenotypic differentiation on the basis of underlying visuo-attentional mechanism.

Next, we examined visual search performance according to mean target detection latency in children with iASD and DS+/-ASD. Superior search performance was observed in both iASD and DS + ASD cohorts when compared to children with DS-ASD. While children with DS + ASD were differentiated from their peers with DS-ASD according to increased levels of non-verbal intellectual disability, these differences remained when non-verbal intellectual ability was taken into consideration. Further, in all cases, autistic trait severity was significantly negatively associated with search latency. This finding suggests that idiopathic forms of ASD and manifestations of ASD in DS share a common visuo-attentional feature: enhanced search performance. While this result requires replication, particularly as sample sizes were small, it implies that idiopathic forms of ASD and comorbid cases in children with DS may share common genetic risk factors and/or neuropathogenetic mechanisms. For instance, we know that certain genes located on chromosome 21 have been implicated in the emergence and expression of iASD (e.g., BTG3, CXADR and NCAM2; Molloy, Keddache, & Martin, 2005). Comorbidity in DS may therefore be the result of the increased genetic dosage of common risk variants. Alternatively, different genetic risk factors may converge at the level of pathogenetic mechanism to produce similar visuo-attentional profiles and phenotypic outcomes in children with iASD and DS + ASD.

This finding prompts a number of conceptual considerations and novel testable hypotheses. Firstly, as superior search performance has been implicated early in the emergence of the iASD phenotype (Cheung et al., 2018; Gliga et al., 2015), we might expect to observe this visuo-attentional strength in infants with DS who later go on to receive clinical diagnosis of ASD. Prospective longitudinal research is necessary to determine whether or not enhanced search performance constitutes an early risk marker for ASD in children with DS.

Secondly, enhanced search performance in iASD has been theorised to manifest on account of early disruption to the development of the alerting system (Keehn et al., 2013; Joseph et al., 2009). This, in turn, is considered to prompt the emergence of an overly focused attentional style, enabling stimulus features to be processed more efficiently at the locus of attention. Support for this proposal comes from research by Blaser, Eglington, Carter, and Kaldy (2015). They examined visual search performance and pupillary responsivity in toddlers with iASD and chronologically age-matched neurotypical controls. According to their results, task-evoked pupillary dilation was greater in toddlers with iASD. As pupillary dilation is considered by many to be a sensitive index of arousal and attentional engagement (Hess & Polt, 1960; Jackson & Sirois, 2009; Kahneman & Beatty, 1966), the authors concluded that superior search performance in iASD reflects a highly focused visuo-attentional style. In light of the current results, it would be interesting to test whether search performance in children with DS + ASD elicits a similarly elevated level of pupil dilation to imply a shared pathogenetic mechanism.

As autistic trait expression in iASD was found to vary significantly according to both FAC and visual search performance, we examined the relationship between them. Contrary to the notion of a common underlying mechanism, no significant effects were observed. One might reasonably argue that the current sample was too small, and that this correlation analysis was underpowered – however, if there is a visuo-attentional profile in iASD, one might expect high correlations between these measures, and therefore the absence of any effect is notable. In reference to the literature, this suggests that children with iASD who excel at visual search are not necessarily those who show heightened irregularity in attentional disengagement, as measured by the gap-overlap task. Finally, we were interested in whether these eye-tracking variables (FAC and search latency) contributed independently to variability in phenotypic expression in children with iASD. We found that FAC, and not search latency, accounted for a significant proportion of the variability in children’s total SRS-2 scores, implying that the gap-overlap task is a more sensitive measure of phenotypic expression in children with iASD.

In conclusion, we present a first insight into the visuo-attentional features associated with ASD comorbidity in DS with reference to iASD and DS-ASD comparison groups. Further, this is the first study to investigate the relationship between performance on gap-overlap and visual search tasks in reference to phenotypic expression in iASD and DS cohorts. An additional merit of this work is that it includes children with low-functioning iASD; despite a 24-fold increase in the number of published papers relating to ASD in the past 30 years (Chakrabarti, 2017), individuals who are severely affected by the phenotype are typically neglected from the literature (Jack & Pelphrey, 2017; Stedman, Taylor, Erard, Peura, & Siegel, 2019). In the face of ongoing clinical and empirical uncertainty surrounding the nature and validity of ASD diagnoses in DS, we find preliminary support for a common visuo-attentional marker (i.e., enhanced visual search performance). Small sample sizes are a limitation and replication with larger participant samples is required. Nevertheless, this study highlights the need to progress, as a field, beyond the use of behavioural measures of phenotypic description that are limited in their sensitivity and to focus empirical efforts instead on more fine-grained behavioural measures, like eye tracking, to tackle enduring questions about the nature of proposed neurodevelopmental comorbidities.

## CRediT authorship contribution statement

**Jennifer M. Glennon:** Conceptualization, Methodology, Investigation, Formal analysis, Visualization, Writing - original draft, Writing - review & editing. **Hana D’Souza:** Supervision, Writing - review & editing. **Luke Mason:** Software, Data curation. **Annette Karmiloff-Smith:** Conceptualization, Supervision. **Michael S.C. Thomas:** Supervision, Writing - review & editing.

## Declaration of Competing Interest

None.

## Footnotes

1 Greenhouse-Geisser values are presented as the assumption of sphericity was not met (Mauchly’s W=.53, *p*=.002), yielding an adjustment to the degrees of freedom (df).
